# Stroke Core Volume Weighs More Than Recanalization Time for Predicting Outcome in Large Vessel Occlusion Recanalized Within 6 h of Symptoms Onset

**DOI:** 10.3389/fneur.2022.838192

**Published:** 2022-02-21

**Authors:** Noemie Ligot, Sophie Elands, Charlotte Damien, Lise Jodaitis, Niloufar Sadeghi Meibodi, Benjamin Mine, Thomas Bonnet, Adrien Guenego, Boris Lubicz, Gilles Naeije

**Affiliations:** ^1^Department of Neurology, CUB Hôpital Erasme, Université Libre de Bruxelles (ULB), Brussels, Belgium; ^2^Department of Radiology, CUB Hôpital Erasme, Université Libre de Bruxelles (ULB), Brussels, Belgium; ^3^Department of Interventional Neuroradiology, CUB Hôpital Erasme, Université Libre de Bruxelles (ULB), Brussels, Belgium

**Keywords:** large vessel occlusion, ischemic core, perfusion CT, within 6 h of onset, outcome

## Abstract

**Introduction:**

Current guidelines suggest that perfusion imaging should only be performed > 6 h after symptom onset. Pathophysiologically, brain perfusion should matter whatever the elapsed time. We aimed to compare relative contribution of recanalization time and stroke core volume in predicting functional outcome in patients treated by endovascular thrombectomy within 6-h of stroke-onset.

**Methods:**

Consecutive patients presenting between January 2015 and June 2021 with (i) an acute ischaemic stroke due to an anterior proximal occlusion, (ii) a successful thrombectomy (TICI >2a) within 6-h of symptom-onset and (iii) CT perfusion imaging were included. Core stroke volume was automatically computed using RAPID software. Two linear regression models were built that included in the null hypothesis the pre-treatment NIHSS score and the hypoperfusion volume (Tmax > 6 s) as confounding variables and 24 h post-recanalization NIHSS and 90 days mRS as outcome variables. Time to recanalization was used as covariate in one model and stroke core volume as covariate in the other.

**Results:**

From a total of 377 thrombectomies, 94 matched selection criteria. The Model null hypothesis explained 37% of the variability for 24 h post-recanalization NIHSS and 42% of the variability for 90 days MRS. The core volume as covariate increased outcome variability prediction to 57 and 56%, respectively. Time to recanalization as covariate marginally increased outcome variability prediction from 37 and 34% to 40 and 42.6%, respectively.

**Conclusion:**

Core stroke volume better explains outcome variability in comparison to the time to recanalization in anterior large vessel occlusion stroke with successful thrombectomy done within 6 h of symptoms onset. Still, a large part of outcome variability prediction fails to be explained by the usual predictors.

## Introduction

The anterior circulation of the human brain is provided by the anterior cerebral arteries (ACA) and middle cerebral arteries (MCA) that emerge from the internal carotid arteries (ICA). It is responsible for the irrigation of 422 out of the 541 cm^3^ of each hemisphere, including most of the motor and eloquent cortices (1). This explains why anterior circulation large vessel occlusions (LVO) account for over sixty percent of the morbidity and mortality related to stroke (2). Anastomoses between left and right anterior circulations, between posterior and anterior circulations through the Circle of Willis, as well as between leptomeningeal collaterals, may maintain a temporary blood supply to the brain territories affected by LVO. The effectiveness of those anastomoses delimitates both the ischemic core, defined as the critically hypoperfused tissue with low cerebral blood flow (CBF) that will die regardless of subsequent reperfusion status, and the ischemic penumbra, defined as the “tissue at risk” that is potentially salvageable and has the capacity to recover if reperfusion is achieved (3).

In LVO, if recanalization therapy is feasible within 6 h of symptoms onset, there is a class I recommendation against performing brain perfusion imaging to select patients that may or may not benefit from thrombectomy. As the dictum goes “time is brain”, and so a direct transfer to the angiography suite is favored over any delay needed for perfusion imaging acquisitions (4). On the other hand, if a patient with LVO presents between 6 and 24 h after symptom onset, recanalization is warranted only when perfusion parameters are deemed favorable (5, 6). However, given the underlying pathophysiology, perfusion analysis should give the same information whether imaging is done within 6 h of symptom onset or thereafter. Furthermore, amounting evidence shows that collateral status analysis supersedes the Time to Reperfusion (TTR) in predicting outcome from recanalization therapy (7, 8). Similarly, the HERMES collaboration that pooled patient-level data from all randomized controlled trials that compared endovascular thrombectomy with standard medical therapy in patients with an anterior circulation ischaemic stroke demonstrated that TTR mattered mostly for low stroke ischemic core volumes (9). Yet, actual recommendations argue against computed tomography perfusion (CTP) imaging in early onset LVO on the basis of: (1) the delay incurred in further imaging acquisitions, (2) the risk of potential overestimation of the core volume in early LVO (Ghost effect) (10) reported in as many as 50% of cases (10, 11), (3) the technical issues that make CTP unreliable in as many as 13% of cases (12). Still, the increasing number of patients treated by mechanical thrombectomy for LVO and the associated costs of the procedure plead an accurate selection of patients who will most benefit from the procedure within 6 h of symptom onset. Conceptually, despite its potential limitations, perfusion imaging still stands as a major contributor to recanalization therapy decision.

The aim of this study is to assess the relative importance of TTR and core volume estimated by the RAPID software in the outcome prediction of patients with a LVO occlusion presenting within 6 h of symptom onset with successful recanalization therapy. To do so, a regression analysis was done on a large monocentric cohort of patients to weigh the yield of perfusion analysis and TTR in early onset LVO.

## Methods

### Population

The studied population is derived from the stroke registry of Erasmus Hospital in Brussels (Belgium) where all case of acute stroke since January 2015 are recorded and our analysis included patients admitted between January 2015 and June 2021 (13, 14). Inclusion criteria were patients presenting with (i) age over 18 years-old, (ii) acute anterior LVO, defined as an occlusion of the ICA (T-type), MCA (M1 or M2 segments) and ACA (A1 or A2 segments) (iii) successful recanalization defined as a reperfusion score of Thrombolysis in Cerebral Infarction (TICI) > 2a, within 6 h of symptoms onset and (iv) perfusion CT imaging done within 6 h of symptom-onset with reliable analysis by Rapid software.

### Imaging

Pre-interventional imaging included non-contrast CT, CT angiography and CT perfusion (CTP). Ischemic core was defined as brain volume with CBF under 30% of the CBF of the homologous zone in the contralateral hemisphere. Ischemic penumbra was defined as brain volumes where the T_max_ of contrast product arrival exceeded 6 s. Those volume were automatically computed with the Rapid software (15) on which the cut-offs were based on (6).

### Outcomes

The primary outcome measure was functional outcome, using (1) the National Institutes of Health Stroke Scale (NIHSS) score at 24 h post-thrombectomy, and (2) a good clinical outcome at 90 days defined as a Modified Rankin Scale (mRS) score ≤ 3.

### Statistical Analysis

#### Variable Definition and Regression Models

Variable of interest were selected based on previous stroke outcome predictive models: Age in years (16), NIHSS at admission (17, 18), ischemic core (9) and time to recanalization (TTR) (19) were chosen as covariates in a linear regression model to predict NIHSS 24 h post-recanalization. The aforementioned variables were chosen as covariates in a logistic regression model to predict a favorable stroke outcome at 90 days. The models' ability to predict favorable stroke outcomes were assessed by calculating the area under receiver operating characteristic curves (AUC) of sensitivity vs. (1-specificity). AUC is used to measure how well a model correctly classifies patients into two groups of favorable outcome or not. An AUC of 0.5 corresponds to a prediction that is no better than chance where 50% of patients would be assigned to each group, a higher AUC means better classification, and an AUC of one corresponds to perfect classification.

To assess the relative weight of ischemic core volume and TTR, admission NIHSS and age–both unmodifiable intrinsic patients stroke characteristics–were included in the *null model* as nuisance parameters.

#### Correlations

Pearson rank correlations were used for correlation between explanatory and outcome variables. Corrected *p*-values for multiple comparisons by Bonferroni was set at 0.017.

#### Ethics

The study was reviewed and approved by the Ethics Committee of Erasmus Hospital, Brussels, Belgium. Written informed consent for participation was not required for this study in accordance with the national legislation and the institutional requirement.

#### Data Availability

Data can be shared upon reasonable request.

## Results

### Population

We identified 102 successful LVO recanalizations occurring in patients who underwent thrombectomy within 6 h of symptom onset among the 377 thrombectomies realized in the period considered (Table 1). Reliable CTP analysis by the Rapid software was available for 94 of these patients who thus constituted the study cohort. Mean age was 71 ± 12 years. Mean admission NIHSS was 15 ± 7. The rest of the clinical characteristics are summarized in Table 1.

**Table 1:** Clinical characteristics.

Age (mean ± SD, years)	71 ± 12
tPA (*n*, %)	62 (69)
Occlusion location:	
T-ICA (*n*, %)	23 (29)
M1 (*n*, %)	54 (64)
M2 (*n*, %)	24 (28)
RAPID data analysis (mean ± SD)	
CBF >30% vol	26 ± 36 ml
Tmax >6 s	100 ± 64 ml
Mismatch ratio	13.4 ± 14.2
Mismatch volume	74 ± 50 ml
NIHSS Score (mean± SD)	
Before therapy	15 ± 7
24h after therapy	9 ± 8
Difference	−5 ± 8
Recanalisation time from onset	217 ± 99
mRS score 90 days (*n* = 62)	
0 (*n*, %)	8 (13)
1 (*n*, %)	14 (23)
2 (*n*, %)	9 (15)
3 (*n*, %)	10 (16)
4 (*n*, %)	4 (6)
5 (*n*, %)	1 (2)
6 (*n*, %)	14 (23)

*SD, Standard Deviation; tPA, tissue Plasminogen Activator; ICA, Internal Carotid Artery; M1, M1 division of the Middle Cerebral Artery; M2, M2 division of the Middle Cerebral Artery; CBF, Cerebral Blood Flow; NIHSS, National Institutes of Health Stroke Scale; mRS, modified Rankin Scale*.

### Correlation Analysis

**Figure 1:**
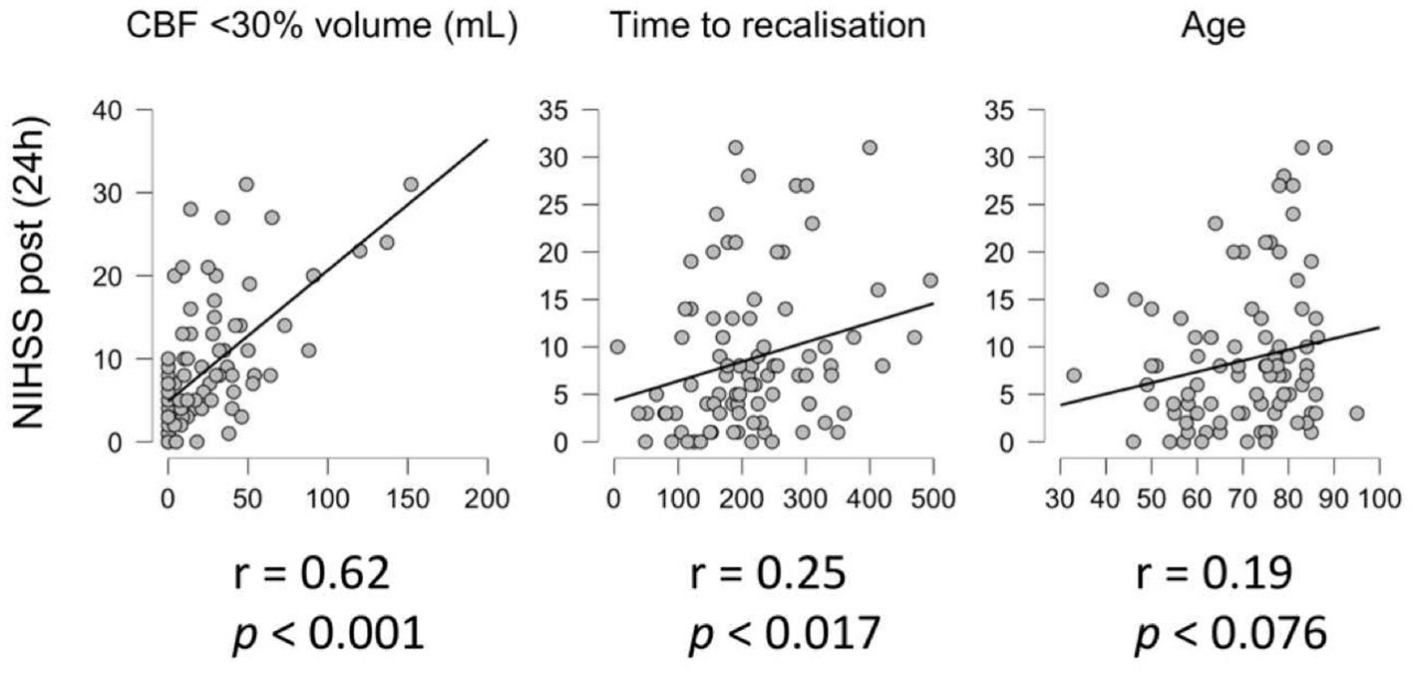
Correlation analysis. Correlation between NIHSS 24 h post treatment and core volume (CBF < 30%, left), time to recanalization (center) and age (right).

There was a significant correlation between ischemic core volume (CBF <30%) and NIHSS at 24 h post treatment (*r* = 0.62, *p* <0.001) and between TTR and NIHSS at 24 h post treatment (*r* = 0.25, *p* =0.017), but not between patients' age and NIHSS at 24 h post treatment (*r* = 0.19, *p* = 0.076) (Figure 1).

### Linear Regression Model for NIHSS 24 h Post Treatment Prediction

Table 2, illustrates the results of the linear regression model. This multiple linear regression model was statistically significant to predict NIHSS 24 h post treatment (*R*^2^ = 0.57, *F*_(4, 82)_ = 27.2, *p* < 0.001) and explained 57% of the variability of the NIHSS 24 h post-treatment (Nagelkerke determination coefficient, *R*^2^ = 0.57). In this model core volume and the TTR individually accounted for 17 and 3.5% of the outcome variability respectively when NIHSS on admission and age were included in the *null model* as nuisance parameters (20).

**Table 2:** Linear regression coefficients for the variables assessed in the prediction model.

	**Coefficient**	**95% CI**	***p-*** **value**
Age	0.13	−0.12 0.178	0.086
Admission NIHSS	0.36	0.21 0.57	< 0.001
Time to recanalization	0.19	0.04 0.28	0.012
CBF < 30%	0.46	0.08 0.16	< 0.001

*CI, confidence interval; NIHSS, National Institutes of Health Stroke Scale; CBF, Cerebral Blood Flow*.

### Linear Regression Model

**Figure 2:**
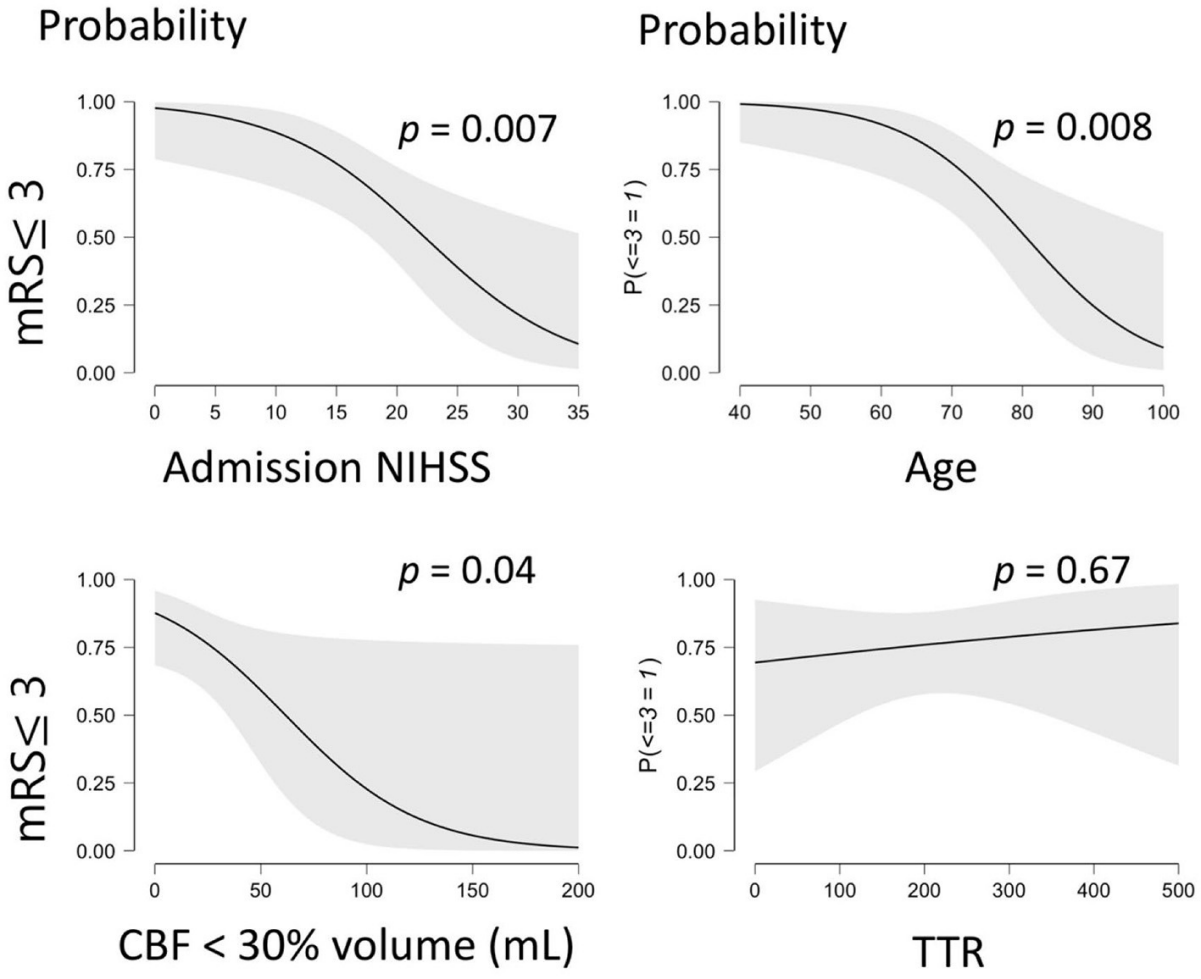
Illustrates the probability of a favorable outcome (mRS ≤ 3) being associated to significant predictive variables. mRS, modified Rankin Scale; CBF, Cerebral Blood Flow; TTR, Time to Recanalization. The gray shaded area on each graph corresponds to the 95% confidence interval.

Figure 2 illustrates the amendable variables associated to a favorable stroke outcome. A logistic regression was performed to weigh the effects of the core volume and TTR on the likelihood that participants have a favorable stroke outcome (mRS ≤ 3) at 90 days.

Table 3 shows the respective odd ratios and associated p-values for the variables assessed in the prediction model. This logistic regression model was statistically significant to predict favorable outcomes (*R*^2^ = 0.57, χ^2^(61) = 32.97, *p* < 0.001) with a model AUC of 0.9 (sensibility: 90%, specificity: 62%).

**Table 3:** Odd Ratios of the variables assessed in the prediction model.

	**Odds ratio, [95% CI]**	***p***
Higher admission NIHSS	0.85 [−0.29–0.043]	0.008
Older age (years)	0.99 [−0.2–0.03]	0.007
Higher CBF <30% volume	0.97 [−0.06–0.00]	0.049
TTR	1.02 [**–**0.06 0.09]	0.66

*CI, confidence interval; NIHSS, National Institutes of Health Stroke Scale; CBF, Cerebral Blood Flow; TTR, Time to Recanalization*.

This model explained 57% of the variability of the favorable stroke outcome (Nagelkerke determination coefficient, *R*^2^ = 0.571) with 14% (*R*^2^ = 0.142) of the variability explained by the core volume and 0.6% explained by the TTR (*R*^2^ = 0.006) when NIHSS on admission and age were included in the *null model* as nuisance parameters (20).

## Discussion

The main findings from this study are 2 fold. First, the ischemic core volume is more effective than TTR to predict both early and late favorable outcomes following successful LVO recanalization within 6 h of symptoms onset. Second, 40% of the variability of favorable outcomes fail to be apprehended by canonical predictors.

The findings of this study, albeit limited by its monocentric nature, are likely to be generalizable to other populations of LVO treated by thrombectomy within 6 h of symptoms onset. Indeed, the cohort matches closely to the characteristics of the pooled populations in the meta-analysis that included the five main trials that validated mechanical thrombectomy (MR CLEAN, ESCAPE, REVASCAT, SWIFT PRIME, and EXTEND IA) in terms of age, admission NIHSS, rate of ICA and M1 occlusion (21). Likewise, patients in our cohort achieved recanalization faster but within a similar timeframe (216 vs. 285 min) than in the validation studies, and displayed a similar rate of favorable outcome (mRS≥3: 67 vs. 64%) (21).

Over the past years, many predictions models have been proposed to isolate factors associated with favorable stroke outcomes (22). The generalizability of such models is always controversial as no single model is likely to address all situations, subgroups and local contexts. However, a common finding prior to the rise of endovascular thrombectomy (EVT), regardless of anterior LVO, was that simple clinical variables (e.g., the Six Simple Variables including age, pre-stroke functional status, living alone pre-stroke, being able to walk unaided, lift both arms off the bed and have a normal verbal Glasgow Coma Scale score) were found to be efficient in predicting independent survival after stroke (16, 23–25). The addition of imaging data like CT or diffusion-weighted magnetic resonance imaging (MRI) stroke volume (26) to clinical data does not seem to improve prediction accuracy (27).

In EVT, the Stroke Prognostication Using Age and NIHSS (SPAN) index developed for fibrinolysis therapy (17), displayed a similarly fair prognostic discrimination (18) that was improved by adding the weighted relative contribution of age and NIHSS at admission (multiplied by three) (28). Our study also highlights age and NIHSS at admission as main predictors of favorable outcome. However, those two canonical parameters only account for 40% of the variability found in favorable outcomes measured at 24 h and 90 days. Furthermore, TTR is a significant predictor for an early favorable outcome with only a marginal contribution to the prediction model accuracy, whereas it is not significant for a late favorable outcome. Thus, TTR is not a main contributor to LVO outcome within 6 h of symptoms onset.

Brain perfusion assessment in LVO < 6 h of symptom onset could, therefore, be relevant if we want to single out patients that will really benefit from EVT. Indeed, in LVO < 6 h of symptom onset, there is no significant mismatch to be found between the ischemic core and the penumbra in between 38 and 64% of cases, which sheds some doubt about the benefit/risk ratio and the cost-effectiveness of EVT in those patients (29, 30). Such lack of a significant mismatch and a large ischemic core both independently predict poor outcomes in LVO treated by EVT < 6 h of symptom onset (29, 30). In our study, we focused on patients who benefited from successful recanalization therapy < 6 h of symptom onset to get a more accurate vision of the predictive value and weight of the ischemic core on a favorable outcome. The association between core volume and a favorable outcome in our cohort was both significant and relevant: the ischemic core volume in itself explained almost 20% of favorable outcome variability that was not accounted for by age or NIHSS. This fact shows that CTP in LVO < 6 h onset is important to predict a favorable outcome, much more so than TTR. This association between favorable outcome and core volume is also supported by CT angiogram collaterals status assessment studies. Indeed, collateral status is tightly associated to ischemic core volume (31) and shows a similar association with functional outcome regardless of the time from symptoms onset (32).

Still, in our model, 40% of outcome variability remains to be explained by other parameters. A ghost effect overestimating the stroke ischemic core volume could explain part of the model imprecision. However, while a ghost effect is found regularly in LVO, the mean overestimation is around 10 ml (10), which seems too small comparatively to the 140 ml sylvian artery perfusion volume to be responsible for significant effects (1). Thus, the remaining unpredicted favorable outcome variability must be found elsewhere. Predictive factors could come from intrinsic patient clinical variability or from the occurrence of complications. Indeed, the morbidity and mortality following a stroke are primarily related to cerebral complications in the first week, followed by medical issues from the second week onwards (e.g., infections, pressure sores, thrombo-embolic events), both of which play an increasing role in modeling the outcome (33–38). While this hypothesis is worth further investigation, the fact that the model, as it is, explains the same amount of variability for early and late favorable outcome argue for a potential role of other acute cerebral compensatory mechanisms/failures that escape the conventional work-up. Indeed, several parameters not assessed by automated softwares like *Rapid*, such as venous outflow assessment from acute stroke angio-CT (39) or the quantitative lesion water uptake calculated from non-contrast head CT (40), have been shown to be associated with favorable outcomes. Similarly, treatment related parameters beyond TTR can provide prognostic information such as the luxury perfusion reflected by early venous filling after EVT that predicts early favorable outcome (13). These neuroimaging markers may provide an insight into the physiological effects following LVO that is both patient-specific and dynamic, and may thus complement other known clinical variables in predicting functional outcome.

## Conclusion

Core stroke volume better explains outcome variability in comparison to the time to recanalization in anterior large vessel occlusion stroke with successful thrombectomy done within 6 h of symptoms onset. Still, a large part of outcome variability prediction fails to be explained by the usual predictors suggesting that further research for more accurate acute stroke outcome biomarkers is warranted to identify which patients would benefit the most from thrombectomy within 6 h of symptoms onset.

## Data Availability Statement

The raw data supporting the conclusions of this article will be made available by the authors, without undue reservation.

## Ethics Statement

The studies involving human participants were reviewed and approved by Ethics Committee of Erasmus Hospital, Brussels, Belgium. Written informed consent for participation was not required for this study in accordance with the national legislation and the institutional requirements.

## Author Contributions

NL, SE, and GN: study design, data collection, analysis, and manuscript writing. CD, LJ, NS, BM, TB, AG, and BL: data collection and drafted manuscript for intellectual content. All authors contributed to the article and approved the submitted version.

## Funding

GN is a Postdoctorate Clinical Master Specialists at the FRS-FNRS (Brussels, Belgium).

## Conflict of Interest

The authors declare that the research was conducted in the absence of any commercial or financial relationships that could be construed as a potential conflict of interest.

## Publisher's Note

All claims expressed in this article are solely those of the authors and do not necessarily represent those of their affiliated organizations, or those of the publisher, the editors and the reviewers. Any product that may be evaluated in this article, or claim that may be made by its manufacturer, is not guaranteed or endorsed by the publisher.
